# Strategies for implementing the interRAI home care frailty scale with home-delivered meal clients

**DOI:** 10.3389/fpubh.2023.1022735

**Published:** 2023-01-23

**Authors:** Lisa A. Juckett, Haley V. Oliver, Govind Hariharan, Leah E. Bunck, Andrea L. Devier

**Affiliations:** ^1^Occupational Therapy Division, School of Health and Rehabilitation Sciences, College of Medicine, The Ohio State University, Columbus, OH, United States; ^2^Coles College of Business, Kennesaw State University, Kennesaw, GA, United States; ^3^Lifecare Alliance, Columbus, OH, United States

**Keywords:** evidence-based practice, home-and community-based care and services, evaluation, nutrition, implementation science, knowledge translation, pragmatic trials

## Abstract

**Introduction:**

Frailty is a complex condition that is highly associated with health decline and the loss of independence. Home-delivered meal programs are designed to provide older adults with health and nutritional support that can attenuate the risk of frailty. However, home-delivered meal agencies do not routinely assess frailty using standardized instruments, leading to uncertainty over the longitudinal impact of home-delivered meals on frailty levels. Considering this knowledge gap, this study aimed to facilitate home-delivered meal staff's implementation of a standardized frailty instrument with meal clients as part of routine programming. This article (a) describes the use of Implementation Mapping principles to develop strategies supporting frailty instrument implementation in one home-delivered meal agency and (b) examines the degree to which a combination of strategies influenced the feasibility of frailty instrument use by home-delivered meal staff at multiple time points.

**Methods and materials:**

This retrospective observational study evaluated staff's implementation of the interRAI Home Care Frailty Scale (HCFS) with newly enrolled home-delivered meal clients at baseline-, 3-months, and 6-months. The process of implementing the HCFS was supported by five implementation strategies that were developed based on tenets of Implementation Mapping. Rates of implementation and reasons clients were lost to 3- and 6-month follow-up were evaluated using univariate analyses. Client-level data were also examined to identify demographic factors associated with attrition at both follow-up time points.

**Results:**

Staff implemented the HCFS with 94.8% (*n* = 561) of eligible home-delivered meal clients at baseline. Of those clients with baseline HCFS data, staff implemented the follow-up HCFS with 43% of clients (*n* = 241) at 3-months and 18.0% of clients (*n* = 101) at 6-months. Insufficient client tracking and documentation procedures complicated staff's ability to complete the HCFS at follow-up time points.

**Discussion:**

While the HCFS assesses important frailty domains that are relevant to home-delivered meal clients, its longitudinal implementation was complicated by several agency- and client-level factors that limited the extent to which the HCFS could be feasibly implemented over multiple time points. Future empirical studies are needed to design and test theoretically derived implementation strategies to support frailty instrument use in the home- and community-based service setting.

## Introduction

Home-delivered meal programs provide community-dwelling older adults with health and nutritional support to optimize wellness and reduce the need for more advanced healthcare services (1, 2). Programming typically targets older adults who are unable to safely and independently perform routine mealtime activities (e.g., shopping, meal preparation), who live alone and below the poverty line, and experience fair-to-poor health (3, 4). In a recent nationwide sample from the United States, 76% of home-delivered meal clients had at least one activity of daily living (ADL) impairment, 74% had five or more reported health conditions, and 33% experienced difficulty affording food items on a routine basis (5).

The aforementioned characteristics of home-delivered meal clients also place them at elevated risk for *frailty* (6–8). Operationally defined, frailty is a recognizable state of marked vulnerability resulting from age-related declines in physiological health (9). While the term “frailty” used to be synonymous with “disability,” more refined definitions of frailty have recently emerged with greater consideration for the accumulation of physical, social, and cognitive factors that contribute to physiological decline (10, 11). As defined by Fried at al., presence of three of the following five criteria are indicative of frailty: low grip strength, low energy levels, slowed walking speed, low physical activity, and unintentional weight loss (9, 12). Frailty-related health declines drastically minimize older adults' ability to tolerate health stressors (e.g., acute illness), leading to poorer health outcomes and individual healthcare costs that can total over $30,000 annually (13, 14). Globally, up to 27% of community-dwelling older adults experience frailty (10)–15% in the United States—with frailty being more prevalent among women, racial and ethnic minorities, and people of lower income (15).

Although trained health professionals (e.g., physicians, nurse practitioners) can address frailty and its associated risk factors (16), the assessment of frailty can be time- and resource-intensive (17, 18), particularly with older adults, such as home-delivered meal clients, who present with complex needs and chronic comorbidities (3, 19). Given that over 70% of home-delivered meal clients experience frailty (20), innovative approaches are needed to regularly assess and monitor the frailty levels of older adults enrolled in home-delivered meal programs. Routine evaluation of frailty can alert home-delivered meal staff of changes in frailty levels, which may warrant additional services or interventions. Timely intervention can potentially reverse the severity of frailty, thereby reducing clients' risk of further decline and institutionalization (21).

Frailty has previously been assessed in the home-delivered meal setting by means of secondary data analyses (e.g., chart review) (15) but has yet to be examined longitudinally through the use of standardized frailty instruments administered directly to clients. The implementation, also referred to as “uptake” or “use,” of such instruments by home-delivered meal staff has the potential to provide home-delivered meal agencies with metrics representing clients' improvement or maintenance of frailty levels—metrics that are necessary for demonstrating the valuable impact of these meal programs overtime (22, 23).

Despite the high prevalence of frailty and the importance of monitoring frailty levels, there is little guidance for how home-delivered meal staff members can effectively implement frailty instruments, particularly when those instruments are implemented at multiple time points (e.g., baseline, 3-month, and/or 6-month follow-up). Accordingly, the purpose of this paper is to (a) describe the use of Implementation Mapping (24) principles to develop strategies supporting frailty instrument implementation in one home-delivered meal agency and (b) examine the degree to which a combination of implementation strategies influenced the feasibility of frailty instrument use by home-delivered meal staff at multiple time points. Insights from agency staff and leadership also illuminate challenges and opportunities for implementing frailty instruments within the home-delivered meal context. This work underscores practical considerations for how home-delivered meal providers may assess frailty and continuously monitor health status changes among a highly vulnerable group of community-dwelling older adults.

## Materials and methods

### Study design

To evaluate our implementation strategies, we used a retrospective observational design and examined home-delivered meal staff's implementation of the interRAI Home Care Frailty Scale (HCFS) (25) at baseline (meal program enrollment), 3-months, and 6-months.

### Setting

The agency partner for this study was a not-for-profit organization that provided home-delivered meals and nutritional support services to older adults, age 60 and over, in the five surrounding counties of Columbus, Ohio. With a staff of over 200 full- and part-time individuals, our partner agency employed a diverse group of staff members representing the fields of social work, nursing, community health, and dietetics, as examples.

### Frailty instrument description

The HCFS is a 30-point scale developed from a secondary analysis of client-level interRAI Home Care data (25). HCFS items cover the following five domains: *function, movement, cognition and communication, social interaction*, and *nutrition*, and it also assesses the presence of common clinical conditions (e.g., renal failure, pneumonia), with higher total HCFS scores indicating greater levels of frailty. Agency staff members and leaders, in collaboration with our research team, selected to implement the HCFS given its perceived ease of use by clinical and non-clinical staff, implementability via telephone, and evidence of acceptable criterion-related validity (average Kappa values for each domain: *function* = 0.59; *movement* = 0.32; *cognition and communication* = 0.51; *social interaction* = 0.28; *nutrition* = 0.20) (25). Unlike the agency's standard in-take assessment that was implemented with clients upon enrollment and every 12-months thereafter, staff implemented the HCFS at 3-month and 6-month follow-up points for the purposes of the present study. At all time points, the HCFS was administered via telephone as agency staff were not permitted to complete in-home assessments with clients given COVID-19 restrictions.

### Implementation mapping

**Figure 1:**
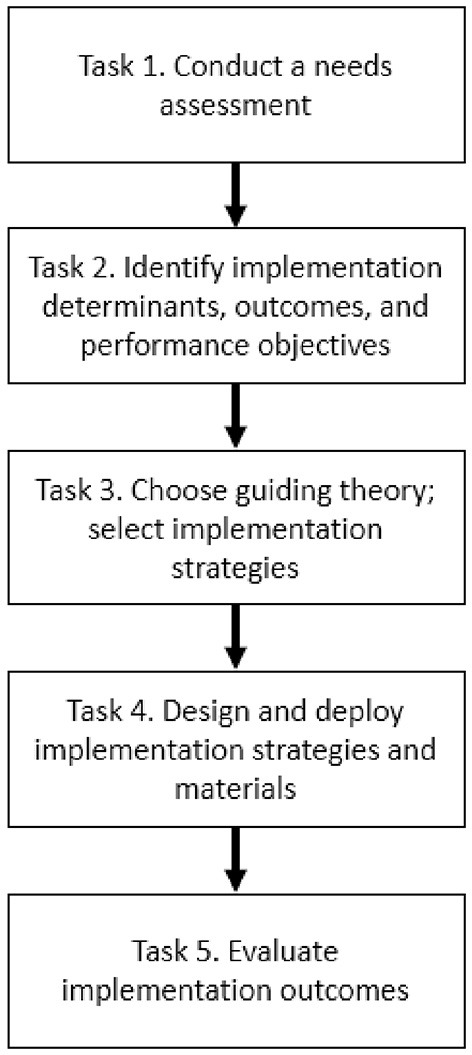
Implementation mapping steps informed by Fernandez et al. (24).

Implementation Mapping is a systematic, theory- and evidence-informed process designed to guide the development of *implementation strategies*—or the approaches used to support the uptake of high-quality interventions, assessments, programs, or practices (24, 26). It consists of a series of tasks that culminate in implementation strategy deployment and the evaluation of implementation of outcomes (e.g., feasibility, adoption, fidelity) (27). The manner in which these tasks were applied to HCFS implementation is described below and expands upon prior methods used to develop implementation strategies in the community-based setting (28). All Implementation Mapping tasks (Figure 1) were co-led by agency partners (assistant director of nutrition programs, a case manager, and three administrators) in collaboration with our research team.

### Task 1. Conduct a needs assessment

Our needs assessment was conducted in two phases. Phase 1 involved 1-on-1 interviews and focus groups with home-delivered meal staff as well as personal care assistants, homemakers, nurses, and dietitians employed by our partner agency. Interview and focus group guides were structured to evaluate the factors (i.e., determinants or barriers and facilitators) influencing evidence-based practice implementation in the context of home- and community-based services more broadly. Qualitative data underwent directed content analysis to identify key determinants of evidence implementation, and complete methodological details are reported elsewhere (29). In Phase 2, we held 3, 1-h meetings with agency leadership and staff to understand current workflow procedures and how those procedures may be altered as a result of implementing the HCFS with home-delivered meal clients.

### Task 2. Identify implementation determinants, outcomes, and performance objectives

Through our needs assessment, we identified that perceived determinants at the agency-level—rather than policy-level, staff-level, or client-level—served as major determinants of HCFS implementation. In recognition of these determinants, home-delivered meal program directors, assessment staff, and the research team established the target outcomes (27) and performance objectives that needed to be achieved in order for HCFS implementation to be successful. Establishing target outcomes and performance objectives also informed the research team's selection of data sources available within the agency that were needed for our outcome evaluation.

### Task 3. Choose guiding theory; select implementation strategies

The identification of perceived determinants (Task 2) was informed by the Consolidated Framework for Implementation Research (CFIR)—a meta-theoretical framework of constructs representing the dynamic context within which organizations may implement new practices (30). Thus, the CFIR also guided the research team's selection of HCFS implementation strategies that were vetted and confirmed by agency partners. Strategies were drawn from the Expert Recommendations for Implementing Change (ERIC) taxonomy (31) using the CFIR-ERIC matching tool (32). Whereas, the CFIR provides uniform nomenclature to define implementation barriers and facilitators, the ERIC taxonomy is a compilation of over 70 implementation strategies hypothesized to promote the uptake of evidence-based practices into routine care. The CFIR-to-ERIC matching tool uses expert opinion data to generate a rank-ordered list of specific strategies to support evidence-based practice implementation.

### Task 4. Design and deploy implementation strategies and materials

Our team began designing our implementation strategies and materials over the course of 5-months prior to HCFS implementation. Strategy development was led primarily by the agency's assistant director of nutrition programs as well as our research team. All strategies were designed and operationalized according to recommendations by Proctor et al. (26). These recommendations include: clearly identifying the individuals involved in providing (actors) and receiving each strategy (action targets), describing how the strategy is delivered (action), and establishing the strategy's main goal (outcome), justification (rationale), and frequency (temporality, dosage).

### Task 5. Evaluate implementation outcomes

To evaluate HFCS implementation outcomes, data were collected retrospectively from our agency's custom HCFS documentation website from the 12-month time period of June 1, 2020–May 31, 2021, as per chart audit recommendations for implementation studies (33). HCFS data were examined monthly by the research team to determine rates of HCFS implementation for individual clients at baseline, 3-months, and 6-months. Rates were established by calculating the proportion of clients who had documentation of the HCFS being completed compared to the total number of clients eligible for the HCFS. Documented reasons why staff were unable to complete the HCFS with clients were analyzed descriptively. We also conducted univariate analyses to assess staff member's rates of implementation at all three time points, and used bivariate analyses to identify demographic characteristics associated with client attrition at 3-months and 6-months. All associated research activities were approved by the Institutional Review Board at The Ohio State University (#2020E1238).

## Results

### Results from task 1: Conduct a needs assessment

**Figure 2:**
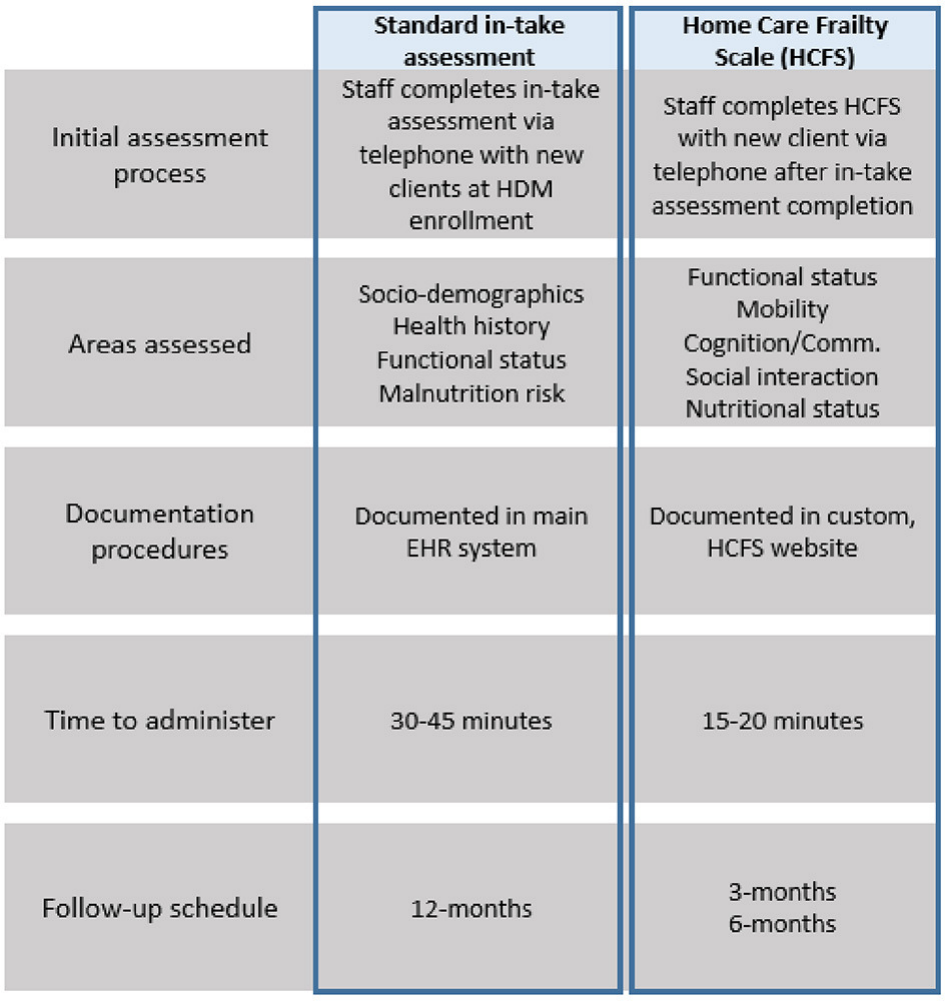
Comparison of standard in-take assessment with HCFS at baseline, 3-month, and 6-months; HDM, home-delivered meals; EHR, electronic health record.

Our needs assessment found three key, agency-level determinants influencing the implementation of evidence-based practices in the home-delivered meal setting. These determinants, as defined by the CFIR (30), were: (1) networks and communications, (2) available resources, and (3) compatibility (29). *Networks and communications* referred to the nature and quality of how HCFS data were documented and shared within the agency; *available resources* included the time, staff, and equipment needed to implement the HCFS; and *compatibility* referred to the perceived “fit” of the HCFS with the agency's existing workflow, standard practices, and values. Our meetings with agency leadership and staff also allowed our team to gather robust understanding of standard in-take assessment procedures and the extent to which these procedures would be altered by implementing the HCFS at multiple time points. Figure 2 compares staff's processes of implementing both the standard in-take assessment and the HCFS.

### Results from task 2: Identify implementation determinants, outcomes, and performance objectives

Determinants of implementation were identified through the completed needs assessment (see above). Consensus from agency leadership and staff indicated their primary outcome of interest was staff's *feasibility* of implementing the HCFS longitudinally. For the present study, feasibility was defined as the utility or suitability of an evidence-based innovation for everyday use, which can be measured through the collection and analysis of administrative or health record data (27, 34). Lastly, agency partners identified the following, single performance objective for staff: To implement the HCFS with 100% of home-delivered meal clients—funded through Title-IIIC—at baseline as well as 3-months and 6-months after program enrollment, for all clients still enrolled in a meal plan.

### Results from task 3: Choose guiding theory and select implementation strategies

Identified determinants from the CFIR, recommendations from the CFIR-ERIC matching tool, and input from agency leadership and staff informed our selection of five implementation strategies to address the determinants of networks and communications, available resources, and compatibility. These included (a) conduct ongoing training, (b) identify and prepare a HCFS champion, (c) complete pilot testing, (d) change record systems, and (e) perform chart audits and provide feedback.

### Results from task 4: Design and deploy implementation strategies and materials

The five implementation strategies designed and deployed by our team are described below and specified in (Table 1).

**Table 1:** Specification of strategies to promote HCFS implementation.

**Specification criteria**	**Implementation strategies**				
	**Conduct ongoing training**	**Identify and prepare a HCFS champion**	**Complete pilot testing**	**Change record systems**	**Perform chart audits and provide feedback**
Actor	Research team	Research team	Research team	Research team, HCFS champion, IT department	Research team
Action	Conduct initial and follow-up training sessions	Prepare internal staff member to serve as on-site HCFS resource and liaison to research team	Conduct HCFS with HDM clients prior to full HCFS roll-out	Develop a web-based portal to increase ease of HCFS documentation	Review rates of HCFS adoption
Action target	HDM assessment staff	Assistant director of nutrition programs	HDM assessment staff	IT department; documentation systems	HDM assessors
Dose	1-h in-person training; 1-h online training	2-h review of administration and documentation of HCFS	1-month pilot of staffing administering the HCFS with HDM clients	Development of the 30-item web-based HCFS	Monthly chart review of completed HCFS
Temporality	Initial in-depth training in Jan 2020; follow-up training in June 2020; as needed emails and phone calls	Bi-weekly phone calls with research team; monthly phone calls with research team and assessors	7 HDM assessors completed up to 10 HCFS with clients	Initial development of web-based HCFS; modifications to web-based HCFS made after pilot testing was complete (Jan 2020)	Every month for the first 3-months of implementation
Outcome	HCFS feasibility	HCFS feasibility	HCFS feasibility	HCFS feasibility	HCFS feasibility
Justification	CFIR-ERIC matching tool; agency input	CFIR-ERIC matching tool; agency input	CFIR-ERIC matching tool; agency input	CFIR-ERIC matching tool; agency input	CFIR-ERIC matching tool; agency input

Table adapted from Proctor et al. (26) recommendations for specifying implementation strategies. HCFS, Home Care Frailty Scale; HDM, home-delivered meal; IT, information technology. CFIR, Consolidated Framework for Implementation Research; ERIC, Expert Recommendations for Implementing Change.

### Conduct ongoing training

When developing the structure for HCFS staff training, our team purchased the HCFS training manual ($65) (35), which contained instructions for how to administer and interpret each of the 29 HCFS items. We then converted the training manual into a presentation format that was delivered to home-delivered meal staff members during an initial training session. Initial training consisted of an in-depth review of all HCFS items, examples of how to score the HCFS, demonstration of how to document the HCFS (see “Change record systems” description below), and a question-and-answer session. Five months after initial training, a 1-h follow-up “booster” training session was held. Training materials were updated with additional examples of how to administer and interpret client responses to individual HCFS items. Staff were also provided a “cheat sheet” document for interpretation and scoring of item responses.

### Identify and prepare a HCFS champion

The agency's assistant director of nutrition programs held the role of HCFS champion. In addition to their leadership within the agency, the champion had extensive knowledge of agency workflow and oversaw assessment procedures completed by staff. In this role, the HCFS champion received advanced training in administering and interpreting the HCFS, facilitated our research team's receipt of monthly HCFS data files for auditing and analysis, and maintained a tracking log of clients to indicate when each baseline HCFS was completed as well as anticipated dates for 3-month and 6-month HCFS collection. Each month, the HCFS champion emailed staff the updated tracking log and also sent weekly emails containing a list of clients due to have their HCFS completed. Staff were responsible for administering the HCFS within a 14-day window of clients' estimated 3-or 6-month HCFS follow-up date. Moreover, the champion held biweekly phone calls with our research team to discuss concerns with HCFS implementation and to clarify discrepancies in staff's interpretation of individual HCFS items.

### Complete pilot testing

After initial HCFS training, home-delivered meal staff (*n* = 7) pilot tested the HCFS with a minimum of 10 clients over a 30-day period. Piloting the HCFS allowed for home-delivered meal staff and leadership to gain comfort with its administration and allowed our research team to clarify any challenges with HCFS interpretation prior to formally rolling out the HCFS with all home-delivered meal staff and clients. Results from pilot testing also informed how our research team structured “booster” training sessions with staff, such as by including specific examples of how to interpret/score responses to each HCFS item.

### Change record systems

As part of our agency's routine operating procedures, all standard in-take assessments were completed by staff and entered into the agency's main EHR system. Building HCFS items into the main EHR required involvement from programmers external to the agency, thus complicating the extent to which staff could feasibly document the HCFS electronically. As a solution to this documentation issue, the agency's information technology (IT) department developed their own HCFS website that allowed staff to document HCFS data electronically but separately from the agency's EHR system. Staff accessed the HCFS website to enter the following data: (a) client ID, (b) date HCFS was attempted, (c) HCFS completion [yes/no], (d) reason if HCFS was *not* completed [unable to reach client after three attempts, client unenrolled from services, client deceased, client on hold, etc.], (e) responses to all 29 HCFS items, (f) date follow-up HCFS was expected, and (g) name of staff member completing the HCFS.

### Perform chart audits and provide feedback

The HCFS champion shared monthly data sets with our research team who monitored the extent to which staff completed the HCFS at baseline, 3-month, and 6-month time periods. Implementation rates were reported to agency leadership during each monthly team call. When rates of implementation fell below 60–70%, the HCFS champion provided additional reminders to staff via email and encouraged staff to share any challenges they experienced relative to HCFS use or scoring. Further details on this audit-and-feedback approach and our four additional implementation strategies are described in Table 1.

### Results from task 5: Evaluate implementation outcomes

#### Feasibility of implementation

**Figure 3:**
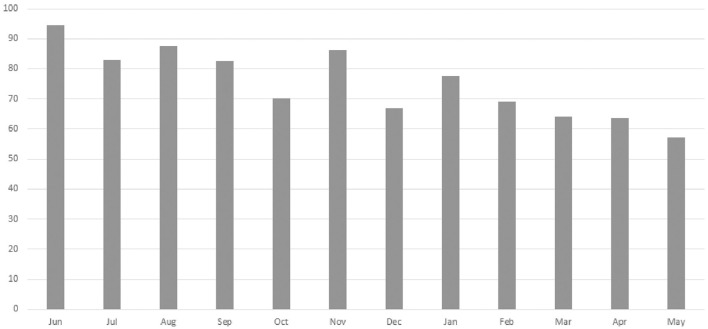
Monthly rates (%) of Home Care Frailty Scale implementation (June 2020–May 2021).

A total of 13 staff members implemented the HCFS with home-delivered meal clients between June 2020–May 2021 with higher rates of implementation noted among staff members with the most years of service to our partner agency (Table 2). Analyses from our retrospective chart review indicated that rates of implementation were highest in June 2020 (94.6%) and lowest in May 2021 (57.1%) (Figure 3). Staff completed the HCFS with 94.8% of eligible clients at the baseline timepoint. Of those who completed the HCFS at baseline, however, staff were only able to obtain HCFS data from 43% of clients at 3-months and 18% of clients at 6-months.

**Table 2:** Agency assessment staff and rates of Home Care Frailty Scale implementation at baseline, 3-months, and 6-months.

	**Baseline** **(%)**	**3-month** **(%)**	**6-month** **(%)**	**Years of service**	**Position with agency**	**Professional background**
Assessor 1	11.2	12.9	-	< 1 year	Community and client manager	Psychology
Assessor 2	22.6	16.7	8.3	3 years	Client coordinator	Social work
Assessor 3	30.8	24.6	30.6	39 years	Dining center coordinator assessor	Community health
Assessor 4	26.0	27.9	27.8	20 years	County coordinator	Community health
Assessor 5	4.3	6.3	5.6	2 years	Client and community liaison	Community health
Assessor 6	0.2	0.4	0.9	< 1 year	RDN	Dietetics
Assessor 7	-	0.4	3.7	10 years	Client and community liaison	Community health
Assessor 8	-	3.3	4.6	3 years	Driver supervisor	Social work
Assessor 9	-	1.7	-	< 1 year	Intern	Dietetics
Assessor 10	0.2	-	-	8 years	Asst. Director of Nutrition Programs	Social work
Assessor 11	-	-	1.9	< 1 year	Intern	Dietetics
Assessor 11	0.2	-	-	33 years	Registered Nurse	Nursing
Assessor 13	0.2	0.4	-	< 1 year	Intern	Dietetics

Note: 3.2% unknown at baseline (*n* = 18); 5.7% unknown at baseline (*n* = 13); 7.4% unknown at 6-months (*n* = 8).

**Figure 4:**
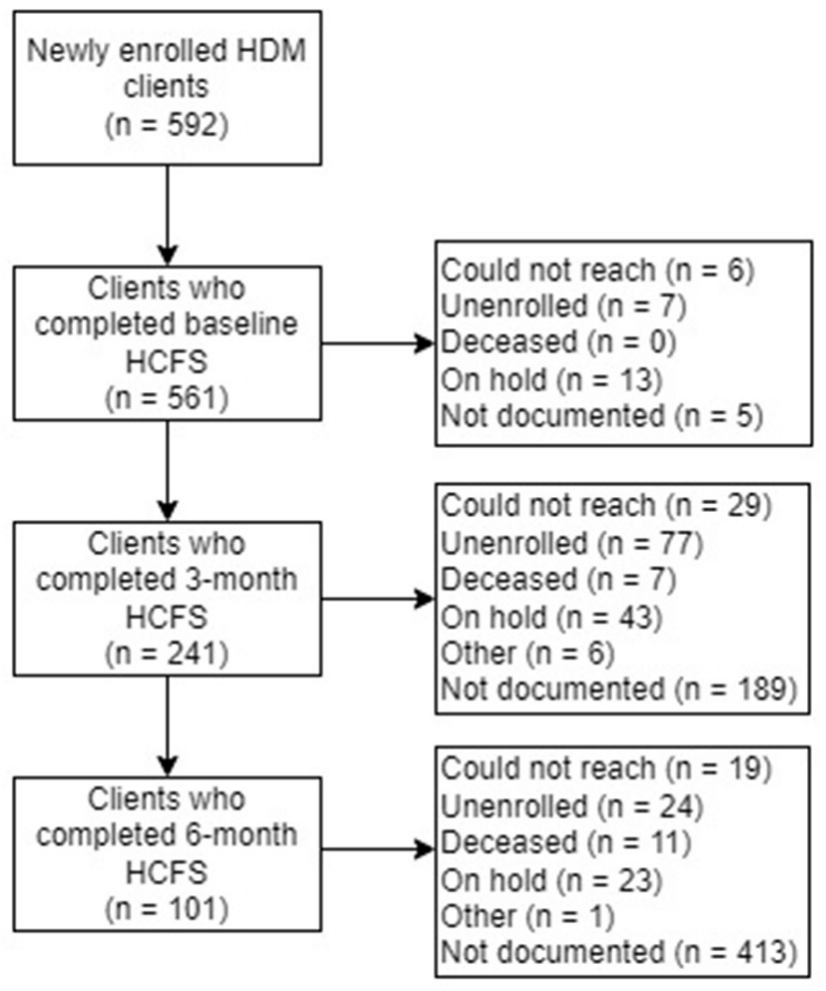
Home-delivered meals (HDM) clients who completed the Home Care Frailty Scale (HCFS) at baseline, 3-months, and 6-months.

At baseline, the most common reason staff were unable to complete the HCFS was attributed to clients (*n* = 13) being “on hold” as they were recently hospitalized or admitted to a care facility (e.g., rehabilitation facility), had a family member who could temporarily provide nutritional support, or were in the process of relocating. At 3-month and 6-month follow-up, clients having “unenrolled” from their meal plan was the most frequently *documented* reason staff could not complete the HCFS (*n* = 77 at 3-months; *n* = 24 at 6-months). Overwhelmingly, however, staff did not or were not able to document the reasons clients were lost to follow-up (Figure 4).

At the client-level, while age was not associated with baseline or 3-month HCFS completion, 6-month attrition was significantly more likely among older clients (*p* < 0.01). Relatedly, there was no significant difference by race between those who completed and those who did not complete the baseline or 3-month HCFS; however, there was a significant difference at the 6-month time point in that clients who identified as African-American or black were more likely to complete the 6-month HCFS compared to those who identified as white (*p* = 0.04). Lastly, clients living with relatives were significantly more likely to complete the baseline HCFS (*p* = 0.03) as compared to clients who indicated an “other” household composition. There were no significant differences in HCFS follow-up based on gender, marital status, or county of residence (i.e., rural-urban residence).

Clients who completed the HCFS at 3-months but not 6-months had significantly higher 3-month total HCFS scores (*p* = 0.02). In other words, those with greater frailty at the 3-month time point were significantly less likely to complete the 6-month HCFS (Table 3). Table 4 presents the average baseline, 3-month, and 6-month HCFS scores by domain, including total HCFS scores. With the exception of the social interaction domain score at the 6-month time point, all domain scores and total HCFS scores indicated a downward trend—or improvement in frailty—based on available data.

**Table 3:** Demographic characteristics of clients who completed the baseline home care frailty scale (HCFS) (*n* = 561).

	***n***	**%**
**Gender**		
Female	321	57.2
Male	237	42.2
Did not answer	3	0.5
**Race** ^*^		
African-American or Black	134	23.9
Black	409	72.9
White	1	0.2
Native American or Alaskan	6	1.1
Alaskan	3	0.5
Asian	5	0.9
Other	3	0.5
Declined to answer		
**Ethnicity**		
Hispanic	3	0.5
Non-Hispanic	558	99.5
**Marital status**		
Married	161	28.7
Widowed	163	29.1
Single	104	18.5
Divorced	105	18.7
Separated	15	2.7
Partnered	2	0.3
Declined to answer	11	2.0
**Household composition** ^*^		
Lives alone	276	49.2
Lives with relatives	108	19.3
Lives with spouse	146	26.0
Lives with non-relative	13	2.3
Other	8	1.4
Did not answer	10	1.8
**County of residence**		
Franklin (urban)	396	65.8
Champaign (rural)	26	4.6
Madison (partially rural)	60	10.7
Logan (rural)	25	4.5
Marion (rural)	54	9.6
Age^*a^	75.0 (*M*)	10.1 (*SD*)
3-month HCFS completed^*^	241	43.0
3-month missing	320	57.0
6-month HCFS completed	101	18.0
3-month missing	460	82.0

^*^Indicates statistically significant (*p* = 0.05) association with 3-month or 6-month HCFS completion.

^a^Values for “age” presented at mean and standard deviation.

**Table 4:** Home care frailty scale (HCFS) scores at baseline, 3-month, and 6-month time points by domain.

	**Baseline**		**3-month**		**6-month**	
	***n*** = **561**		***n*** = **241**		***n*** = **101**	
	**Mean**	**SD**	**Mean**	**SD**	**Mean**	**SD**
Function domain	3.22	2.03	3.08	2.04	2.85	1.59
Movement domain	1.71	1.13	1.57	1.09	1.54	1.08
Cognition and communication domain	1.0	1.58	0.91	1.50	0.71	1.33
Social interaction domain	1.30	1.48	1.21	1.45	1.35	1.46
Nutrition domain	0.57	0.94	0.52	0.98	0.27	0.68
Total HCFS score	8.44	4.72	7.87	4.86	7.25	3.70

## Discussion

This study examined one home-delivered meal agency's process of implementing the HCFS—an instrument for measuring the frailty levels of home-delivered meal clients at multiple time points. Although our strategies to support HCFS use by home-delivered meal staff were systematically developed using principles from Implementation Mapping (24, 28), our strategies did not lead to the feasible collection of HCFS data from clients overtime. Though staff demonstrated a high rate of HCFS implementation at baseline (94.8%), our findings also indicated major challenges to collecting follow-up HCFS data at 3-month and 6-month time points for reasons such as staff being unable to contact clients, clients having meal deliveries placed on temporary “holds,” or clients becoming unenrolled from services. Our analyses also indicated that certain demographic factors, particularly older age, race, and household composition, were associated with attrition at either the 3-month or 6-month time point. Importantly, clients with greater frailty scores (or worse frailty) at 3-months were significantly less likely to complete the 6-month HCFS, potentially indicating that clients with greater 3-month HCFS scores may be more vulnerable to health decline and in need of additional services or interventions, though these findings require further investigation to understand reasons for attrition.

Beyond factors at the client-level, there were certainly agency-level obstacles that influenced the feasibility of HCFS implementation. Insights provided by our agency partners, described below, shed light on these complex challenges and opportunities for improvement given the importance of monitoring the frailty levels of home-delivered meal clients over time. Specifically, these insights draw attention to the intricate details that may have been overlooked when our agency and research team members were designing strategies to support HCFS implementation. Consistent with our guiding framework, insights are organized by constructs from the CFIR (30).

### Agency insights: HCFS implementation challenges

#### Networks and communications

Perhaps the most significant agency-level factor influencing HCFS implementation was the manner in which it was documented and how HCFS data were communicated across the agency. Given that modifications within the main EHR system could not be made directly by the agency, the agency's IT department built a custom website—accessible only to agency staff—for HCFS documentation. Though this solution was initially a viable option to support HCFS use, it ultimately posed challenges for our team over the course of our study period. For instance, as indicated in Figure 2, both the in-take assessment and HCFS evaluated areas related to functional status and nutrition. These areas of overlap were duplicative and interrupted the flow of staff's assessment procedures, especially as staff were required to access two different documentation systems to complete all in-take and baseline HCFS items. This interruption also hindered staff's natural course of conversation in the first encounter during which staff could build rapport with clients. Further, staff found substantial difficulty in tracking which clients needed to be contacted for their 3-month and 6-month HCFS. Although the HCFS champion's tracking log and reminder emails helped alert staff when they needed to contact clients for follow-up, these reminders did not indicate if clients were still actively receiving meals, nor did they list client phone numbers. Accordingly, staff were expected to log into their EHR system account, verify that the client had an active meal plan, and obtain the client's phone number, increasing the total amount of time staff were expected to dedicate to HCFS follow-up activities.

#### Available resources

Our agency's lack of integrated documentation systems also limited the extent to which HCFS data could be entered longitudinally for individual clients. To accommodate for this barrier, the research team had one dedicated member who was responsible for merging baseline, 3-month, and 6-month HCFS data together for individual clients, but this was not a sustainable solution for tracking client frailty levels. An additional, though minor, barrier to HCFS implementation was staff's lack of access to dual-monitor computers during standard in-take assessments. After completing in-take assessments in the EHR system, staff immediately transitioned to implementing the baseline HCFS with clients. However, given the challenge of logging out of the EHR system, opening a web browser, and accessing the custom HCFS website on the same computer, staff often completed the HCFS on a paper form and entered client responses—at a later time—through the HCFS website. The timing of data entry, though, was occasionally delayed given staff's other demands and work responsibilities.

#### Compatibility

The concept and format of the HCFS were initially perceived to be compatible by our agency's administrative leaders. Despite these perceptions, leaders later expressed their concern that the information collected via the HCFS was being underutilized internally. Once HCFS implementation began, agency staff and leaders quickly learned of the additional needs of clients (e.g., mobility needs, social interaction needs) that were not necessarily being met by meal delivery alone. Our agency partners expressed their discomfort with assessing but not *addressing* these needs that were revealed as a result of implementing the HCFS longitudinally.

### Agency insights: Opportunities to advance HCFS implementation

#### Advancing networks and communications

Given that documentation was arguably the primary barrier to HCFS implementation, integrating the 29 HCFS items directly into the agency's main EHR system could have likely streamlined documentation, particularly during the baseline period where staff completed both the in-take assessment and HCFS. Going forward, centralizing this information in one location has the opportunity to decrease staff burden, improve assessment workflow, and enhance staff's interaction with clients (36). The return to in-person baseline assessments, pending statewide adjustments to COVID-19 restrictions, may also facilitate more streamlined assessments of frailty as staff can leverage their professional judgment and observational skills to determine the extent to which frailty domains (e.g., mobility, ADLs) are impaired (37, 38).

#### Advancing available resources

Notably, staff who implemented the HCFS were partially compensated through a demonstration project grant which reduced the agency's expenditures toward implementation activities. As these funds were temporary, alternative strategies are needed to support staff's future ability to implement the HCFS feasibly and more consistently. Integrating HCFS items into the agency's main EHR system is a first step toward minimizing assessment and documentation burden on staff. Though, while customized changes to EHR systems have shown promise for improving the quality of staff documentation behaviors (39), these system-level changes may need to be augmented by additional sources of support to promote assessment implementation (40). One additional option for this support is through clinical alerts which have served as effective reminders for staff who are involved in client documentation activities (41, 42). These alerts may take the forms of e-mails, electronic “flags” directly within client charts, and/or pop-up notices within the EHR. Such alert systems can also be configured to deliver text message reminders to staff if documentation is not completed for clients on a specified date (43). While these alert systems have led to improvements in documentation, there is also the threat of “alert fatigue” which may negatively impact staff job performance and satisfaction (44). Thus, use of these alerts, how often they are triggered and under what circumstances should be thoughtfully considered in collaboration with staff and leaders involved in documentation procedures (45).

#### Advancing HCFS compatibility

Implementation of the HCFS revealed frailty-related needs (e.g., fall risk factors, mental health concerns) that staff did not feel fully equipped to address. Although home-delivered meal providers can serve as “gatekeepers” to other community-based services and supports for older adults (46), our agency's staff were not sufficiently aware of recommendations and local resources that could be shared with clients who indicated specific frailty needs. Cataloging these resources for staff, prior to the study period, may have facilitated their ability to make recommendations or referrals to other health and nutrition services, thereby improving the “fit” of the HCFS with the mission of our partner agency to maximize older adult health and wellbeing (19, 47).

### Building from our agency insights

Drawing from our experiences and from the existing literature, we have identified several actionable steps to improve HCFS implementation over time. Our first step is to re-evaluate the “fit” of the HCFS within the workflow and available staff of our partner agency. Given that the time required for staff to complete the HCFS with clients ranged from 15 to 20 min, a shorter scale with similar psychometric strengths may be more compatible with our agency's resources. As one example, the FRAIL scale is a validated tool that can predict functional decline in older adults and can be administered over the phone in < 5 min (48, 49), though it has not yet been implemented specifically in the home-delivered meal context. Implementing a shorter, simpler frailty instrument, such as the FRAIL scale, may also enable staff to monitor frailty more frequently, optimizing the completeness of data collection. With our high rate of attrition based on a variety of reasons (e.g., unable to reach clients; clients unenrolling from meal services), an increase in the frequency of frailty instrument administration may provide our partner agency, and other peer agencies, with more robust data that could be used to examine changes in frailty more consistently and identify clients in need of more comprehensive care. Operationally, the implementation of a simpler frailty tool could serve as a supplement to the monthly socialization “check-in” phone calls that our partner agency has previously completed with clients. Frequent monitoring of frailty may be particularly beneficial with clients who live in the more rural counties of our partner agency's service area. For instance, several rural clients receive frozen meals delivered once every 1–2 weeks as compared to urban-dwelling clients who often receive daily-delivered meals. As previously studied, clients who received daily-delivered meal services—and had more regular contact with drivers compared to frozen meal clients–experienced improved health outcomes (e.g., reduced falls; reduced feelings of loneliness) (50, 51), which has implications for rural-dwelling older adults who experience frailty at a higher rate than national estimates (52). Though daily-delivered meals may not be a feasible option for our partner agency to provide to all their rural clients, more frequent check-ins, such as through phone-based screenings of frailty, may help capture the frailty-related needs of these high-risk clients and inform care planning and service delivery. Notably, our analysis did not yield associations between rurality and HCFS attrition, but this may be attributed to clients' closer proximity to the greater metropolitan area of Columbus, Ohio.

To administer a simpler frailty tool, however, we must ensure that our partner agency has the sufficient capacity to do so. In addition to appointing specific staff members to be responsible for tool administration and adjusting electronic documentation systems, staff should have access to initial training as well as ongoing consultations to confirm procedures for administration, documentation, and interpretation of results. The combination of training and follow-up consultations has been previously found effective for supporting the uptake of evidence-based interventions and assessments (53, 54), particularly in the community setting. Lastly, of the available data our partner agency's staff collected from clients beyond May 2021, we identified that clients had unmet needs, especially in the domains of function and mobility, that could not be addressed through home-delivered meal services alone. Thus, our partner agency and research team are currently in the process of developing a data-driven care model that will specifically address the functional- and mobility-related needs of clients at greatest risk for malnutrition.

### Limitations

Although this study makes several, unique contributions to the understudied home-delivered meal context, it is not without limitations. First, our application of Implementation Mapping principles could have been strengthened by selecting a behavioral change theory or adult learning theory to guide predictions about staff's HCFS use. While our implementation strategy selection was informed by the CFIR (30)—a comprehensive implementation framework—frameworks are not explanatory in nature and can rarely help predict relationships among theoretical constructs (55, 56). Second, we also recognize that our strategies only targeted agency-level implementation determinants whereas policy-level and staff-level determinants may have also played an influential role in our implementation efforts. Third, given that this was a retrospective, observational study, we did not conduct an *a priori* power analysis but rather assessed the rate at which the HCFS was implemented with clients who were served over a 12-month time frame, as recommended for studies of implementation that include chart review methodology (33). Fourth, though the HCFS has evidence of acceptable criterion-related validity, additional psychometric properties (e.g., predictive validity, interrater reliability) are unknown (25). Lastly, we did specify our five implementation strategies (Table 1) per expert recommendations, yet more robust details are needed to understand the mechanisms that promoted—or hindered—HCFS implementation (56, 57). In addition to our own future work, we encourage other teams to routinely track their implementation activities to obtain thorough information on the types of implementation activities completed, their purpose, their duration, and the individuals involved (58, 59).

## Conclusion

Home-delivered meal agencies are essential for providing health and nutrition services to a population of older adults at great risk for frailty-related health decline. Frailty instruments, such as the Home Care Frailty Scale (25), can serve as tools to help home-delivered meal staff assess and monitor the frailty levels of their clients. However, prior to adopting such instruments, home-delivered meal providers are strongly encouraged to comprehensively evaluate and address barriers that pertain to the longitudinal electronic documentation of frailty data, the staff and resources needed to implement frailty instruments consistently, and the extent to which instruments “fit” within agency workflow, standard practices, and values.

## Data availability statement

The datasets presented in this article are not readily available because data collected and analyzed for this manuscript are not publicly available as to comply with our partner agency's data sharing policies; however, materials used to deploy our implementation strategies with staff are available upon request made to the corresponding author. Requests to access the datasets should be directed to lisa.juckett@osumc.edu.

## Ethics statement

The studies involving human participants were reviewed and approved by the Institutional Review Board at The Ohio State University. The Ethics Committee waived the requirement of written informed consent for participation.

## Author contributions

LJ conceptualized this study, led study activities, led manuscript development, and performed statistical analyses. HO completed data management and preliminary data analysis activities. GH assisted with developing the study design and developed the approach to data analysis. LB and AD completed project management activities and provided agency insights, as described in the Discussion Section. All authors contributed to manuscript development and approved the final submitted version.
